# Editorial: The Impact of Migration and Resettlement on Health

**DOI:** 10.3389/fpubh.2022.904697

**Published:** 2022-05-11

**Authors:** Rosemary M. Caron, Amanda Rodrigues Amorim Adegboye, Carlos J. Moreno-Leguizamon, Núria Serre-Delcor, William Sherlaw

**Affiliations:** ^1^Department of Health Management and Policy, Master of Public Health Program, College of Health and Human Services, University of New Hampshire, Durham, NH, United States; ^2^Faculty of Health and Life Sciences and Centre for Healthcare Research, School of Nursing, Midwifery and Health, Coventry University, Coventry, United Kingdom; ^3^Health Development in the School of Human Sciences at the University of Greenwich, London, United Kingdom; ^4^Vall d'Hebron University Hospital, Barcelona, Spain; ^5^École des Hautes Etudes en Santé Publique, Rennes, France

**Keywords:** COVID-19, migrant health, refugee resettlement, healthcare access, mental health, physical activity, maternal health, cultural competency

Migration and displacement are growing phenomena globally that can affect the health and development of host countries. Migrants, defined as a continuum (e.g., regular/irregular/seeking asylum/seeking refugee) can present as a susceptible population for the public health system. For example, in relation to environmental health, the risk for lead poisoning among African refugee children who resettle in the United States (US) remains elevated compared to resident children (1). Resettlement can be a contentious issue for some communities who struggle with the capacity to provide essential services to their existing residents never mind newcomers. Examples of challenges include inadequate translation services; unsafe housing; insufficient public transportation; and a lack of employment opportunities. Further, immigration is generally followed by behavioral, lifestyle, and environmental changes that can significantly increase the risk of disease in the early generations of immigrants. Understanding these changes and exploring their structural determinants is a pivotal step toward a better appreciation of immigrant health and the design of culturally sensitive interventions. As these newcomers provide every community with a richness of labor, culture, language, and history that contributes to the “melting pot” spirit of communities, current social, political, and healthcare structures must be reviewed to address the challenges of migration and displacements.

Applying a strict definition, the word immigrant characterizes individuals who have voluntarily or willingly left their countries of origin and legally moved to another country to live. They have some form of resettlement or permission that allows them to work legally. The main reasons for the resettlement are usually related to economic prosperity, career opportunities, a better education, a better quality of life, or a family reunion. It includes a lengthy formal process and required planning, as well as previous knowledge of the local spoken and written language to comply with specific institutional and legal requirements. Therefore, immigrants always begin their journey as migrants. Yet, although they may stay for an extended period in one single country, they do not necessarily end their journey as migrant settlers. It is essential to make that distinction, as language barriers and the ability to engage and to contribute to the local economy plays a major role in their resettlement. Essentially, a sense of belonging, promotion of integration and social inclusion in their new community can have a substantial positive impact on an individual's wellbeing, thus increasing the potential toward better health outcomes due to a broader range of choices. Fundamentally, these transitions can be less dramatic than the new patterns of modern slavery and less traumatic than the violence of forced migration, due to natural disasters, which occur unpredictably. Individuals, in these instances, may encounter themselves alone, in a strange land, within unfamiliar environments and experiencing challenges that are exacerbated by a lack of language skills (2, 3).

The overarching aim of this Research Topic was to gather a collection of independent research papers to advance the understanding of the complex interaction between migration and health by utilizing a range of disciplinary and methodological approaches to explore these intersectoral relationships. This Research Topic also examined how public health issues are managed in a community that excels or struggles with resettling refugees and migrants. The theme was presented as being broad in scope to encourage the discussion of not only the diverse challenges posed by this vulnerable population but also the ability of the community's public health and healthcare infrastructure to effectively manage (or not) these issues. The role of gender, race/ethnicity, age, nationality, pregnancy status, physical environment, and the cumulative nature of the social determinants of health are explored to demonstrate that migrants are a heterogeneous group. Policymakers and researchers need to understand this plurality when planning for communities with diverse populations.

This editorial aims to provide an overview of the key findings of the papers published in the Research Topic on The Impact of Migration and Resettlement on Health. Upon review of the papers, four overarching themes were identified:

## COVID-19 Impact and Racial and Ethnic Discrimination

COVID-19 significantly impacted not only the functioning of healthcare, education, housing, and employment systems globally, but it also exacerbated the health inequities experienced by marginalized populations, especially racial and ethnic minorities, immigrants, and refugees. As a result of the socioeconomic, political, and demographic profile of these vulnerable populations (e.g., economic segregation, overcrowded housing, exclusion from healthcare services, and cultural and linguistic barriers), many are at increased risk of contracting COVID-19 and experiencing significant morbidity and mortality (4–6). Caron and Adegboye (7) examine the COVID-19 through the lens of a syndemic and highlight the interactions among the social determinants of health. The authors propose that a bi-directional relationship exists between the social determinants of health (e.g., employment, education, housing, poverty, healthcare access) and the COVID-19 syndemic thus exacerbating the impact of this novel disease for at-risk populations. An integrated approach is presented which incorporates the Centers for Disease Control and Prevention's Essential Public Health Services framework with the World Health Organization's Health for All scaffold when considering how individual, structural, sociocultural, and socioeconomic factors interact with each other to result in a disparate risk for marginalized populations with respect to contracting and transmitting COVID-19.

The discussion about the disproportionate risk racial and ethnic minorities face in the age of COVID-19 is noted as the US Latinx population have experienced the second highest hospitalization and mortality rate from COVID-19 compared to Black minorities (8). Like other vulnerable populations, the Latinx population has experienced continual discrimination with respect to housing, education, employment, and healthcare access, as well as discrimination from law and immigration enforcement (9–11). Martinez et al. (12) comprehensively describe racism and the different types that have adversely impacted marginalized populations historically with an emphasis on the Latinx population. The authors propose a research agenda that incorporates the use of biobehavioral research to further explore the physiological embodiment (e.g., chronic mental and cardiometabolic disease) of racism and social inequities among the Latinx populations. The purpose of studying this area could inform institutional and behavioral interventions. As the US is acknowledging, openly discussing, and attempting to address racial inequity at a local, state, and national level, the authors conclude with a call to action by which to include the Latinx population in the materialization of racism research.

## Differential Utilization of Healthcare Services

In the US, the use of emergency rooms or services as a form of primary care has been associated with a lack of health insurance, a lack of access to primary care, low socioeconomic status, patient's perception of the severity of health issues, and convenience (13). In Germany, ~20 million people are treated in an emergency service setting with fewer than half being admitted to the hospital (14, 15). Further, one-third of the patients accessing emergency services could be treated by a general practitioner as their health complaint is not life-threatening (16). The use of emergency services among migrants across Europe has been reported to be higher when compared to non-migrants (17). Sauzet et al. (18) explore the utilization of emergency services among first- and second-generation migrants and non-migrants at internal medicine and gynecology services in Berlin, Germany. Based on the analysis conducted, the authors conclude that there is a difference between the utilization needs and purposes of emergency services among this population sector. Factors contributing to these differences could include varying levels of patient expectations and health literacy levels. The authors recommend that the existing healthcare system be re-structured to enable access to healthcare services for a diverse population and that this information be communicated in a way that is understood by a diverse group.

The challenge of healthcare utilization by migrants in their resettled community is often compounded by numerous barriers (e.g., cultural competence, low health literacy, low socioeconomic status, discrimination and racism) (19). Further, different countries that comprise the European Union often have specific regulations about the types of healthcare services migrants can access. For example, Germany, Belgium, and Denmark provide access to emergency services while Spain provides guaranteed healthcare to all residents and due to reporting requirements in other countries, migrants are dissuaded from accessing health care altogether (20–22). Serre-Delcor et al. (23) examine the health needs and barriers experienced by migrants resettled in Spain when they attempt to access health services. The authors conducted a perception-based cross-sectional survey among social and healthcare professionals who provide care to recently arrived migrants and noted three key findings. First, these professionals perceive new migrants as arriving with worse health status, poor mental health status, and are less likely to misuse the healthcare system when compared to the native population. Second, health professionals perceived a lack of adequate understanding of migrant health rights and recommend that recently arrived migrants be better informed of their rights when in their host country. Third, the main barriers identified by health professionals when working with the migrant population included language and cultural competence issues. The authors conclude by calling for more evidence-based research in this area to help inform health professionals who work with this vulnerable population.

It is important to note that the major disparities in integration policies around the world reflect the major differences in integration outcomes and attitudes around the world. The integration policies identified by the Migration Integration Policy Index (MIPEX) also shape how immigrants and the public respond to these inequalities. A country's approach to integration strongly influences the public's attitudes and behavior toward immigrants. Integration policies are one of the strongest factors shaping the public's willingness to accept and interact with immigrants (24).

## Health Needs Assessment and Wellbeing

Even prior to the current global COVID-19 pandemic, refugee resettlement has been a stressful process often characterized by social isolation, lack of access to resources, poor language proficiency, economic hardship, and perceived/actual discrimination which can adversely affect one's mental health (25, 26). Research in this area communicates a need to further examine this issue in more depth with respect to gender differences in response to resettlement stress and mental health status. For example, a survey of Afghan refugees who resettled in the US determined that lower stress was experienced by females but not males who reported English proficiency and strong family ties. Males reported higher stress than females concerning acculturation dissonance (27, 28). Due to the dearth of research on gender-specific issues regarding post-resettlement stressors for refugees, Nissen et al. (29) examine gender-specific associations of post-migration stressors with an emphasis on subjective wellbeing and the impact of social support among Syrian refugees resettled in Sweden. The authors report that social and financial hardships experienced post-resettlement have an adverse effect on male Syrian refugees. The authors recommend further research be conducted in this area along with the development of gender-sensitive policies aimed at reducing psychological distress.

## Physical Activity/Overweight/Obesity

Physical activity is a modifiable health determinant that contributes to poor health and mortality globally (30). One's level of physical activity is influenced by gender, race/ethnicity, and education level (31–33). Norwegians are considered relatively active in part due to the accessibility of safe, natural spaces, as well as the value placed on physical activity for good health (34, 35). Calogiuri et al. (36) examine the physical activity habits of recent Italian immigrants to Norway, as well as identifying factors that influence health behaviors among immigrant populations in general. Specifically, the purpose of the authors' work was 3-fold: (1) examine the extent to which recently immigrated Italians to Norway perceived their move as having a positive or negative impact on their physical activity practices; (2) compare the physical activity profile between Italians and Norwegians; and (3) identify differences among the Italian immigrant physical activity profile related to key sociodemographic factors. The authors report that a significant majority of Italian immigrants perceived that they were as active in Norway, if not more so, compared to if they stayed in Italy. Physical activity variables (e.g., sitting time, moderate-to-vigorous physical activity frequency and duration) were not different between the two populations, although the Italians queried reported participating in gym exercise more so than the Norwegian population. Sedentary levels were reported in males with a lower educational level, Italians with higher education levels, and those who lived in less urbanized environments. The authors propose that it is important to study how people who move within the European Economic Area engage with opportunities, the culture, and social factors that influence physical activity with respect to migration.

Obesity is another complex and significant public health challenge (37). Studies have shown that Black women of African heritage residing in high-income countries are more likely to enter pregnancy either overweight or obese compared to their White counterparts (38, 39). Further evidence suggests that engagement in physical activity or healthy diet practices during pregnancy or the post-partum period can prevent excessive gestational weight gain and post-partum weight retention (40–45). Thus, improving these health behaviors in minority populations requires culturally sensitive healthcare interventions that consider factors including access to care, health beliefs and practices (46–48). Moore et al. (49) conducted a systematic review and thematic evidence synthesis of select peer-reviewed databases using the Capability-Opportunity-Motivation Behavioral change theoretical model. The authors report that women's behaviors at this life-stage are influenced by motivational factors (e.g., weight gain being good for a growing baby, safety concerns about exercising during pregnancy). Social norms, including acceptance of a different body shape post-partum and daily fast-food consumption, posed a challenge for these women. Women reported having low self-confidence in their ability to lose weight post-partum. The authors propose that behavior change techniques including social support, implementation of credible resources, and demonstrations may be helpful when supporting lifestyle change.

Further, one's socioeconomic status has been demonstrated to be an important health determinant for obesity. For example, research from developed countries has demonstrated that populations of low socioeconomic status are at an increased risk of obesity compared to their high socioeconomic status counterparts (50, 51). Research also suggests that the change in socioeconomic status that often accompanies the transition for migrants from rural-to-urban settings may lead to an increased risk of obesity (52). Wang et al. (53) examined the Yi Migrants Study to determine the following: (1) whether there was an association between socioeconomic status and the prevalence of overweight and obesity in Yi migrants who immigrated from rural-to-urban environments, and; (2) whether differences in these indicators varied by the age of arrival in their urban community. The authors did identify an age-dependent association between socioeconomic status and overweight and obesity among rural-to-urban Yi migrants. Specifically, for Yi immigrants who were <20 years of age at arrival in their urban community, there was a negative association between a higher socioeconomic status and a decreased risk of overweight and obesity. The inverse was observed for Yi migrants who were older than 20 years of age at arrival in their urban community (i.e., a higher socioeconomic status resulted in an increased risk for overweight and obesity.) The authors, based on the findings, recommend that improvements in health literacy and healthy lifestyle education should be available for rural-to-urban migrants.

## Conclusion

As the articles in this Research Topic demonstrate, resettlement of migrants, immigrants, and refugees is generally followed by behavioral, lifestyle, and environmental changes that can significantly increase the risk of disease in the early generations of these vulnerable populations. Understanding these changes and exploring their structural determinants is a pivotal step toward a better appreciation of immigrant health and the design of culturally sensitive interventions.

This Research Topic promotes the expansion of the migration-health research agenda to factors impacting immigrants and refugees (e.g., dietary acculturation, access and utilization of health care, legal status, lifestyle, socioeconomic and political factors). The articles enclosed aim to advance the understanding of the complex interactions among migration and health to identify the major public health issues encountered by refugees and migrants and how communities effectively manage those issues. We expect that this Research Topic will generate information to support advocacy and/or guide the allocation of resources and development of policies that will enable communities to build the capacity to address public health issues impacting resettled refugees and migrants across the globe.

## Author Contributions

RC developed and wrote the Editorial. AR reviewed the Editorial. All authors approved of the Editorial.

## Conflict of Interest

The authors declare that the research was conducted in the absence of any commercial or financial relationships that could be construed as a potential conflict of interest.

## Publisher's Note

All claims expressed in this article are solely those of the authors and do not necessarily represent those of their affiliated organizations, or those of the publisher, the editors and the reviewers. Any product that may be evaluated in this article, or claim that may be made by its manufacturer, is not guaranteed or endorsed by the publisher.
